# Responding to neurodiversity in the courtroom: A brief evaluation of environmental accommodations to increase procedural fairness

**DOI:** 10.1002/cbm.2239

**Published:** 2022-08-05

**Authors:** Betony Clasby, Brigit Mirfin‐Veitch, Rose Blackett, Sally Kedge, Esther Whitehead

**Affiliations:** ^1^ Department of Sociological Studies The University of Sheffield Sheffield UK; ^2^ Murdoch Children’s Research Institute Melbourne Australia; ^3^ Donald Beasley Institute Dunedin New Zealand; ^4^ Centre for Postgraduate Nursing Studies University of Otago Dunedin New Zealand; ^5^ Registered Psychologist, Dyslexia Foundation of New Zealand Christchurch New Zealand; ^6^ The University of Auckland Auckland New Zealand; ^7^ PGDipLitEd Massey University Palmerston North New Zealand; ^8^ Advocate and Advisory Neurodiversity Community of Practice Ako Aotearoa Thrivable New Zealand

**Keywords:** accommodations, courtroom, intervention, neurodevelopmental, neurodiversity, support

## Abstract

Recent research has highlighted that a high prevalence of young adults who have various forms of neurodivergence come into contact with the criminal justice system. Currently, many courts are not designed to respond to neurological differences often seen in young people who engage with them. The aim of this study was to identify ways to make locality courts more accessible, engaging, and ultimately more responsive to neurodivergence. A panel of neurodivergence specialists reviewed the general district courtroom environment of a new specialised young adult list court in Aotearoa New Zealand to identify potential barriers to accessibility and to highlight areas for improvement. The methodology involved naturalistic observation of a typical morning in the courtroom. We identified a series of recommendations with the potential to improve the court experience and increase access to justice for neurodivergent young adults. This study identified specific need for neurodiversity education and screening within the court environment.

## 1 INTRODUCTION

There is increasing awareness that a large proportion of young adults who come into contact with the criminal justice system have neurological differences which may remain mis‐ or undiagnosed and unsupported during court proceedings, potentially contributing to higher incarceration rates (Foster & Young, 2021). The term neurodivergence covers a variety of neurological differences including traumatic brain injury (TBI), autism spectrum disorder (ASD), foetal alcohol spectrum disorder, attention deficit hyperactivity disorder (ADHD), intellectual disability, communication disorders, non‐traumatic acquired brain injury, and specific learning difficulties (for instance dyslexia, dyspraxia, or dysgraphia). Challenges associated with neurodivergence can present in a variety of areas including attentional capacity, functional skills, memory and learning, psychomotor skills, executive functions, social competence, emotion regulation, impulse control, and academic achievement (Catroppa et al., 2017; Morie et al., 2019).

Young adults with these difficulties may encounter barriers to accessing justice when interacting with the criminal justice system. For instance, those with intellectual disability have been found to have significant difficulty understanding legal terminology and court proceedings (Ericson & Perlman, 2001). There is evidenced potential for confabulation – described as an act of ‘honest lying’ (Brown et al., 2013) – in young adults with foetal alcohol spectrum disorder when criminal justice officials do not alter practice to reflect these differences (Pora v The Queen [2015] UKPC 9, [44–48]). Furthermore, the reliance on oral language within the courtroom puts neurodivergent young adults who find communication challenging at a distinct disadvantage (Edwards et al., 2012), potentially contributing to the increased risk of being sentenced to incarceration (Foster & Young, 2021). All these factors can contribute to a hostile environment for those with neurodivergence in the criminal justice system, leading to a need for reform of the court system.

Article 13 of the Convention on the Rights of Persons with Disabilities (CRPD) outlines the importance of ensuring equal and effective access to justice for those with disability, including through the provision of accommodations where required (UN, 2006). Access to justice has been defined as peoples' effective access to the systems, procedures, information, and locations used in the administration of justice (Ortoleva, 2010). To date, 184 States from across the globe have formally committed to the CRPD human rights treaty (UN, 2021) and have thus accepted that barriers to full participation in society still exist for those with disabilities. When exploring these barriers in the context of the criminal justice system it is important to consider procedural fairness as a core construct (Dorfman, 2017). Procedural fairness refers to the idea that when coming into contact with the justice system, not only do people care about the outcome of their case, but also in the way in which it was handled (Brems & Lavrysen, 2013). Valued criteria include being given the opportunity to participate, judge neutrality, being treated with respect, and trust in the system, which stems from understanding the process (Tyler, 2007). Improving procedural fairness not only increases access to justice, but it has also been found to increase compliance with court orders (Bornstein et al., 2011), and reduce reoffending even amongst more violent offenders (Papachristos et al., 2012).

These factors provide an important foundation to consider when identifying accommodations to support neurodivergent young adults who are attempting to access justice in the courtroom. In New Zealand, these calls for reform have resulted in the development of a specific locality court for young adults, termed Young Adult List (YAL) court. This initiative was developed in response to a large body of research which highlights that neuromaturation continues to occur into a person's mid‐twenties (Johnson et al., 2009), suggesting that their experience in court needs to reflect this difference in developmental trajectory alongside any neurodivergence.

There is a dearth of specific recommendations to improve procedural fairness, of what training should entail, and a lack of formal evaluation which limits the applicability of the accommodations (Turner and Hughes, forthcoming). Reports of best practice exist within the literature but are limited (White et al., 2021); therefore the contribution of neurodivergence experts has the potential to be significant and can be harnessed relatively quickly (Baker et al., 2006). It is essential that criminal justice officials and policymakers have access to specific recommendations and a grounding in evaluation to address this gap in the literature and improve access to justice for neurodivergent young adults, in line with the obligations of the CRPD.

Consequently, the aim of this study was to identify and clearly present ways to make the court environment more accessible, engaging, and ultimately more responsive to neurodivergence amongst young adults to facilitate further evaluative work. During an environmental assessment focus group, a panel of neurodivergence specialists reviewed the general district courtroom environment of a new YAL court to identify potential barriers to accessibility and to generate new ideas to empower neurodivergent young adults to better understand, engage, and participate in the courtroom process. These were submitted in a report to the New Zealand Ministry of Justice for potential implementation and evaluation alongside being presented in this paper.

## 2 METHOD

The Young Adult List (YAL) (The Māori name of the Young Adult List is *Iti rearea teitei kahikatea ka taea*. This name was gifted by the Ngati Toa iwi (tribe) and symbolises that challenges can be overcome with determination). This is a specialised District Court in New Zealand, which has been developed for young adults aged 18–25 years. The initial pilot of the YAL court occurred in the Porirua District Court (Porirua is a city near Wellington, Aotearoa New Zealand's capital city), which is where the data collection session for the environmental assessment focus group took place. The YAL trial began in March 2020, although the Covid‐19 pandemic restrictions delayed its official launch until the 31^st^ of July 2020. An undisguised naturalistic observational visit to the court was undertaken to observe the day‐to‐day procedure of the courtroom and the behaviour of those in attendance. In essence, undisguised observation means that the panel were in plain sight of those in the courtroom and courtroom staff were aware of observers (Price et al., 2017). The visit was carried out over a period of 4 h on a single day in April 2021 by five neurodivergence specialists, with 2 h set aside for observation of the court proceedings. Experts were selected on their knowledge base (such as focal area of neurodivergence and of providing support, interventions, and accommodations) and experience (including clinical, research, and lived experience), and the panel's makeup aimed to provide a broad view of neurodivergence from multiple perspectives.

The panel included a speech and language therapist and court‐appointed communication assistant, clinical psychologist, disability rights academic, neuropsychology academic, and neurodivergence advocate representing a non‐governmental organisation. A semi‐structured guidance sheet detailing courtroom features, relationships, and processes was provided to the experts to provide a foundation from which to identify and recommend neurodivergence specific improvements, such as accessibility, engagement, and understanding. The panel of experts were also provided with information booklets routinely distributed to young adults who attend court, along with an A5 notebook to record anonymised feedback. The panel sat in the public gallery alongside other members of the public in order to reduce the likelihood of interrupting the typical routine during the YAL court. The panel then each independently recorded behaviours suggestive of anxiety, stress, or lack of understanding and their apparent triggers, whilst highlighting potential adjustments that could be made to procedures to reduce any barriers to accessing justice relating to neurodivergence. This information was then compiled by the lead researcher into a table. Following this, the table was again disseminated to the panel in a group session to collect any further feedback or suggestions relating to the barriers to accessing justice related to neurodivergence raised. Furthermore, the table was also disseminated to a key member of the judiciary and criminal justice officials to get their feedback on the feasibility of the suggestions.

## 3 RESULTS

**FIGURE 1:**
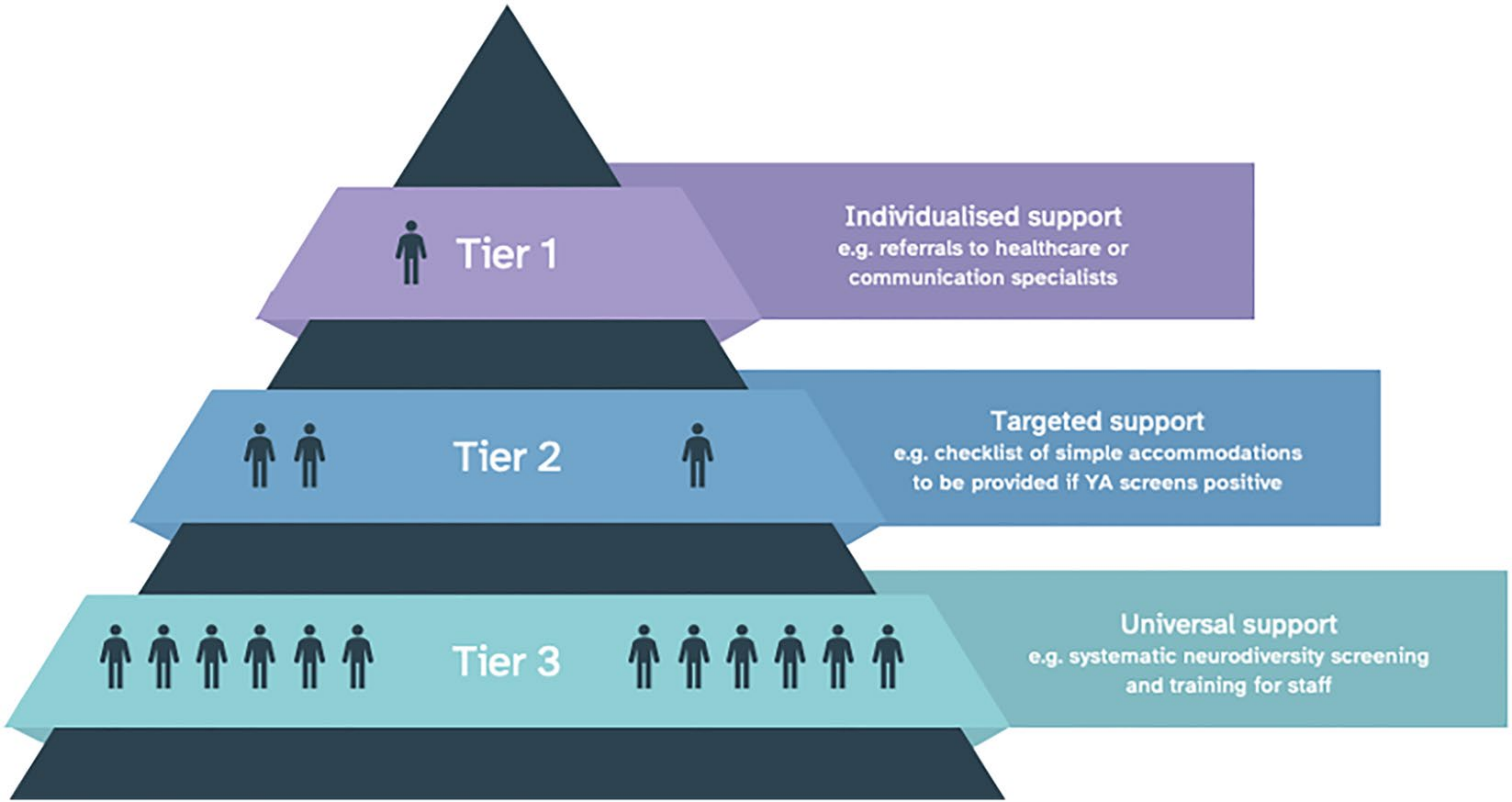
Tiered support model for accommodations provision in the CJS. YA, Young Adult

The panel identified a range of stress‐response behaviours indicative of neurodivergence within the young adults attending court during the time of the observation. The assessors also observed multiple instances of positive practice during the environmental assessment of the YAL which differentiated the courtroom from a typical adult court. This was evident in the number of different stakeholders present in the courtroom, the readability of documentation, design of the courtroom, and involvement across all staffing levels. A number of recommendations were identified as offering potential for greater responsiveness and accessibility to a person with neurodivergence. For clarity and quick reference, specific accommodations, and their staging, are provided in Table 1. The next section shall discuss the key recommendations identified through this process. The accommodations identified fit well and could be implemented in a similar method to the Tiered Support Model framework, which currently describes accommodations offered to students in the education system as being on a continuum of intensity – universal (Tier 1), targeted (Tier 2), or individualised accommodations (Tier 3) (Ministry of Education, 2020). An example of how this could work in the criminal justice system has been presented in Figure 1.

**TABLE 1:** Recommendations

	Universal supports (Tier 1)	Targeted supports (Tier 2)	Individualised support (Tier 3)
Introduction to courtroom	Reflect cultural practices. (e.g., karakia/blessing). Introduce key individuals in court to young adult. Reassure that other individuals are in the courtroom to support other people. Give family/whānau clear direction (or providing a navigator) on where they can sit prior to them entering court.	If the young adult is particularly anxious or has experienced previous trauma, give whānau/family the opportunity to be in a position where they can better support them (e.g. seated beside them).	
Location and physical attributes of the courtroom	Seat young adults next to their defence lawyer/support person as opposed to separately in a dock. Provide a chair for the young adult. Consider removing any dock barriers. Ensure signage is easy read with images alongside wording. Place signage where young adult can see for example, side of chairs, or keep on back for rest of court and give easy read identity label for chest so young adult can see who is who. If possible, reduce number of people in courtroom (particularly in cases where anxiety or sensory difficulties present). A horseshoe layout would be more engaging and less imposing for the young adult. A smaller courtroom. Judges and lawyers should avoid formal clothing.	Provide inflatable seat cushion to assist those with sensory processing and attention issues. Reduce lighting to reduce sensory burden for those who need it. Opportunity to participate via a CCTV camera would be important to provide for highly anxious young adults.	
Engagement with courtroom	Provide means for young adults to engage with the courtroom officials. From a limited resource perspective, this could be through an agreed signal so that young adults can indicate when they want to contribute, when they do not understand, or when they do not feel comfortable talking in front of the court (e.g., green card/red card/blue card to hold up and show their lawyer or the judge discreetly). Give extra thinking time. Responses from the young adults (such as a simple ‘yes’ or nod) may be their attempt to mask a lack of understanding. The young adult may still be processing information or may not have fully understood proceedings. Give praise and encouragement for effort, for asking questions, and for completing tasks. Always try to build on success. If someone has completed all tasks necessary and no longer needs to continue coming to court, acknowledge this accomplishment by asking them what they are going to do next in their life, and congratulate them for starting to turn their life around. Find a way to capture the strengths and motivations of the young adult as these can be used as a motivational tool. This could be completed at screening or by the duty lawyer prior to their court session, if there is time. This allows the young adult to better engage in the courtroom and feel recognised for what they are good at alongside getting help working on aspects which they find more difficult.	Show choices or pathways visually ‐ for example, if you choose A then B will happen. There are often complex processes involved that require young adults to consider many different potential outcomes, and the implications of potential decisions can be hard to understand. These discussions can be much easier if the young adult can 'see' the options laid out on paper, (rather than needing to keep track of being only 'told' the information). A highly relevant choice to show visually would be as follows: for example, follow the guidance of the courtroom's plan for you and *this* will happen, do not and *this* will happen). Present with very clear choices. Have an easy read schedule available for their day at court. This could simply be a paper flow chart with images that the lawyer could notate to show where the young adult is in the courtroom process. As the courtroom is developing a court plan for a young adult, provide this in visual form (preferably with images and minimal written words) so the young adult can see what they have completed and what they have to do and have that available to all while it is being discussed. Include pictures of the court and of the dock in each information booklet if possible. Set of guidelines for courtroom staff and stakeholders to use a range of effective communication strategies that result in active engagement and allow them to check the YA's understanding (not yes and no questions). Avoid saying ‘do you understand?’ as most people will say yes to that question. Instead, say, ‘I've just given a lot of information here, I need to make sure I've explained it properly. Tell me what you understand/tell me what is going to happen next/tell me what you need to do now’. Recap the key points before the young adult leaves and ideally provide them with the key information in written form, for example ‘you probably were following along, but just to make sure, the things you need to do now are…./what we decided today was…../the next thing that will happen is ….’ Spoken and narrated media for the YA would be helpful, so that audio and video options are enabled. Many neurodiverse young adults will prefer access to information, without the need for strong literacy skills, and use alternative modes to access information. Develop a web‐based or app‐based interactive version of booklet in addition to providing a paper‐based copy. Develop easy read interactive webpage detailing what will happen on the day they go to court. Provide other information prior to entry into courtroom for example, create a visual map outside the courtroom to display who does what and how to engage with the process. A TV with a case study in the form of an age‐appropriate animation, could be useful. Make available different modes of information including audio and visual, immediately prior to accessing the court	
Engage with multi‐disciplinary team	Support agencies could follow a co‐location of services model, and then a court coordinator could be employed to liase with the court, young adult, and the multidisciplinary support agencies who go on to provide targeted support for example, in the context of substance misuse. Multi‐disciplinary team available to include: Neurodiversity NGOs; cultural support services; bail support officers; youth community workers; forensic nurses; drug and alcohol specialists; and police prosecutors.	If, during screening, further queries are raised then additional general support may be provided by neurodiversity NGOs. Although formal assessment will be needed to determine most appropriate service.	
Effective communication	Ensure that all courtroom staff and stakeholders use agreed straightforward terminology – try to use as familiar and easy vocabulary as possible. If difficult terminology such as 'discharge without conviction' is used, make sure to explain what it means in lay terms to young adult before they leave court, and is provided in an easy‐read booklet. Speak literally (without similes, metaphors, acronyms, or idioms) for example: o ‘you are free as a bird!’ or ‘results showed she was as clean as a whistle’ (simile = comparison using *like* or *as*). o ‘you can remain at large’ or ‘this case is watertight’ (metaphor = comparison that does not use *like* or *as*). o ‘you are not above the law’ or ‘they are in contempt of court’ (idiom = group of words established by usage as having a meaning not deducible from the individual words alone). Avoid preamble and legalese for example, ‘my learnt friend’. Ensure pace is slower, volume clear, and the content is expressed clearly to reduce barriers to the young adult engaging and participating in the process. Speak directly to the young adult. Adapt communication style according to the needs of each young adult and check in (sensitively) with the young adult to see whether it is working for them. If legal terms are required, then give a brief lay explanation (e.g., released at large, that means you are free to go now).	Give young adults the opportunity to also contribute to court proceedings beforehand by discussing with their defence lawyer what is going to be talked about on their day in court. It would be helpful for their defence lawyer to make sure any plan the young adult is working on is available to look through so they can see what they have completed and what is to be completed, or they can see where they are within the court process and what the next step is. For instance: Today is about putting in pleas, next step is getting reports and then sentencing, etc. These plans (as suggested elsewhere) would be best in easy‐read with images.	Access to communication assistants (see the MOJ's communication assistant quality framework in New Zealand https://www.justice.govt.nz/assets/Documents/Publications/Communication‐Assistance‐Quality‐Framework‐FINAL.pdf).
Wellbeing	Ensure water is available. Try to keep proceedings to a minimum. Some young people may not have access to breakfast/food. Perhaps enquire if there is a local company that could donate/sponsor snacks in the waiting area.	Ask the defence lawyer to raise the option of having fewer people in the courtroom with the young adult beforehand, if sensitive information may be discussed or there is previous trauma. this can be done with a script of verbal prompts given to the duty lawyers to aid this difficult conversation. These suggestions are reliant on trauma‐informed education (understanding how traumatic events can influence current behavioural presentation and de‐escalation) and also the development of a script of verbal prompts (this would only require a few brief simple prompts which could be included in the training to give staff a guide as to how to talk about trauma). Typically people find it a very challenging topic to raise. Provide a space/quiet area/specific room outside of the courtroom to decompress either before or after court. This space should feel calm, have lower lighting and some soft furnishings, where possible.	Develop a response pathway for those who present as anxious/overwhelmed/triggered by seeing police or other professionals. This would require education for staff and stakeholders involved in the response pathway. Be aware that anxiety can manifest as agitation, anger, and frustration and that this is a call for help. It may signal lack of understanding. Anxiety and stress further debilitate comprehension and communication, so minimising stress is the ultimate goal. Remain calm and supportive, regardless of presentation of young adult. Always remember that each and every behaviour is a response to a stimulus (which may not be immediately obvious to the court, but may be very troubling to the young adult). Without the stimulus identified and fixed, the behaviour will not change. Reschedule if needed or hold a closed court. Consider assigning someone in the courtroom to be responsible for checking up on the presentation of the young adult and deciding whether they need assistance or follow up. Targeted training for these assigned individuals would be helpful for them to best identify how to support young adults in the moment by recognising behaviours.
Executive functioning supports	Try to help develop strategies to prevent lost, forgotten, or damaged items. For instance: Provide young adults with important information in both digital (text/email) and paper‐based formats (for example the paper slips given when exiting the courtroom). Courtroom plan support person: Work with young adult to allocate key member from multidisciplinary team or family/whānau to support carrying out administrative challenges relating to courtroom plan, assist with management of paper‐based documents, follow‐ups and reminders for young adult, and scheduling of and help adhering to appointments. Important information should be provided to the young person in multiple formats (text/email/paper‐based), as well as to their allocated courtroom plan support person and other *relevant* parties the young adult consents to.	Ensure the young adult has access to both a computer and in‐person support for writing tasks for example, apology letters. If possible, think of other creative ways of undertaking tasks such as pursuing restorative justice that do not involve writing and are suited to those with theory of mind difficulties.	Proactive advocacy for digitalisation in other agencies for example, Driving Authorities to make processes to gain licence available through an online easy‐read portal and more accessible for those who are neurodivergent.
Training	Training for all courtroom staff and judiciary on neurological difficulties (and neurodiversity broadly), how they may present, factors that might intersect, understanding behavioural presentations, and establishing strategies to support someone who may be struggling. Long term ideally much of this training would be inbuilt in legal education, that of forensic nurses, and other courtroom staff.	Be aware of sensory issues – what they are and why they can be a problem. Try to keep background noise to a minimum. For example, Keys jangling; people eating; doors opening/closing; whispering; rustling paper; lights flickering; too warm/too cold; visually people entering and leaving the court during session. Keep materials out of view when not in use. Try to have a specific place for everything and reduce loose documents. Regarding sensory issues: Utilise the sound systems in place and provide a noise‐cancelling headset/frequency modulation system in each courtroom to help reduce background noise and increase clarity of those speaking.	
Screening	Brief systematic screening for neurodiversity amongst all those who interact with the justice system would help criminal justice workers to identify who may be in need to more targeted, and also more specialised support. This screening would help to frame the different tiers of accommodations and could be carried out prior to engagement with the courtroom; preferably at first interaction with the justice system (e.g. by police). Create a central Internet repository for all neurodiversity guidance, documentation, support maps, information booklets etc., which can be readily accessed for all employees and those attending court. Create a series of easy‐read documents with infographics to support tasks in most common offence pathways. For example, Driving offences > steps to get licence etc.	Specific accommodations could be linked to certain responses on the screening tool, enabling a smoother process of providing more targeted support without requiring this to be fully individualised at this stage.	Referrals to specialist for comprehensive assessment and potential specialist support.

Abbreviations: MOJ, Ministry of Justice; NGOs, non‐government organisations; YA, Young Adult.

## 4 DISCUSSION

### 4.1 Accommodation approaches and recommendations

A variety of accommodations that were recommended by the panel would be beneficial to apply universally for young adults who come into the criminal justice system (at Tier 1). Universal supports were identified as potentially useful in a range of areas such as to increase engagement with the courtroom and to improve wellbeing. Furthermore, the engagement of multi‐disciplinary support agencies would be useful not only in the context of neurodivergence but would take into consideration other important barriers. For instance, lack of responsiveness to young people's ethnicity and culture can lead to even greater barriers for neurodivergent young people. More guidance regarding how to improve communication and language accessibility would be beneficial to inform practice. For instance, it is highly likely that all young adult participants would find clear signposting and ‘comprehension checks’ throughout court discussions helpful. Legal discussions can be highly complex and when legal professionals are talking between themselves, providing alternative and/or additional forms of engagement to verbal discussion is important to encourage an active ‘voice’ during court session. Suggestions as to what format this could take are available in Table 1.

To facilitate effective implementation of many of these accommodations, systemic training would be universally beneficial for criminal justice and enforcement workers. Without adequate training about neurodivergence, courtroom workers are unlikely to be able to alter their practices appropriately. In particular, criminal justice officials need education to develop greater awareness about how neurodivergence may present in a distressing environment, such as a courtroom. Understanding basic information regarding adapting communication to an individual, what ‘plain English’ really means in practice, insight into why people may present with ‘challenging behaviour’, and techniques to support young adults in these circumstances should be included in such education. Including lived experience from neurodivergent young adults who have gone through the system is critical to any education efforts when seeking to improve both staff and young adult perceptions of active participation. Training should be both comprehensive across understandings of neurodiversity, and trauma‐informed; it is important not to solely focus in one area of neurodivergence (for example, autism or brain injury only) over another to avoid misidentification and inappropriate supports. Indeed, young adults in the criminal justice system often present with a myriad of neurological differences, including those related to experience of trauma (Kirby, 2021). In line with this recommendation, researchers have reported improved attitudes amongst criminal justice officers after completing specialist disability awareness courses (Viljoen et al., 2017). However, ensuring the content of these provides a comprehensive, yet engaging view of neurodivergence, which is regularly evaluated with focus groups of young adults and staff members and is critical to ensure practice is evidence‐based.

It is also important to ensure the system can appropriately support staff when improving access to accommodations, and therefore a clear pathway for onward referral is needed so that criminal justice workers have the framework to then provide accommodations. Resources in Easy Read and other accessible formats should be developed for those in common offence pathways to help prevent reoffending (e.g. for driving without a licence, a guidance sheet highlighting the steps to get a licence in an accessible format). All related resources and guidance should be collated and available for both staff and young adults freely online at any stage of their criminal justice trajectory to help support responsive practice.

Systematic screening should be undertaken to assist in the identification of potential barriers to accessing justice and to shape accommodations provided; this is particularly key as neurodivergent difficulties are often not easily identifiable. There should be a clear response pathway to determine what accommodations and support any young adult who screens positive for potential neurodivergence could then receive, including any consideration needed when sentencing. There are clearly evidenced difficulties accessing justice at each stage of interaction with criminal justice system (Foster & Young, 2021). Therefore, screening should be undertaken at first point of contact (i.e., police interaction) and this information should be considered at each step of a young adult's criminal justice trajectory to ensure appropriate access to justice is provided.

Neurodivergence is incredibly heterogenous; a one size approach will certainly not suit everyone (Holness, 2014). For this reason, the provision of targeted supports (Tier 2) would be beneficial for some young people. These supports could be driven by the strengths and challenges of the young adult as identified during screening for neurodivergence. For instance, if a young adult is identified as experiencing high sensory reactivity and associated anxiety on a screening tool, protocol could then dictate that a set of Tier 2 accommodations could be implemented. A systemic pathway to allocate accommodations would also work, considering that many who are neurodivergent have multiple challenges which may need more than one accommodation to achieve active participation (White et al., 2020).

In cases where there is additional complexity, having multi‐disciplinary partners could also provide the opportunity for further specialist support; for example, if an individual has been known to use alcohol to self‐medicate for underlying anxiety and sensory difficulties, then engagement between neurodivergence and substance abuse groups to produce an individualised support plan would be beneficial (Tier 3). This would also include any referral to healthcare professionals for further assessment and specific individualised support needed.

### 4.2 Limitations and future research

There are several limitations of the observational nature of this assessment that are important to disclose. The environmental assessment observation component was restricted to a single point in time (2 h during the morning session on 23 April 2021). This is only a very short time period in this specific court on which to base recommendations, although all attendees had experience of traditional courtroom settings. Courtroom staff were aware that the panel were observing on that day, which may have caused some reactivity bias. In addition, during the observation there was no opportunity to engage with courtroom staff, other stakeholders, or the young adults themselves. The number of assessors that could be included in this exercise was limited; this is a key limitation because the success of the focus group is determined by the composition of the group and the background of its members. Efforts were made to cover a range of different neurodivergent profiles and perspectives, however a critical gap related to the lack of assessors who are Māori, and those with Pasifika backgrounds. Further work led by Māori and Pasifika peoples should be conducted to determine further accommodations that could be recommended in the context of cultural responsivity. Additionally, the group of assessors were all female and most of those appearing that day before the court were male. The group also did not reflect the demographic that the court intends to serve, and it would be highly useful to include young adults and their whānau who have lived experience of court processes as assessors in future panels. Furthermore, due to the prospective and exploratory nature of this work, it was not within the scope of this project to fully implement and evaluate the accommodations recommended. Designing complex evaluation studies for translational research may seem off‐putting for many due to the challenges involved with using large scale experimental designs in real‐life settings.

However, there are ways to undertake effective evaluations, and these should be prioritised. For instance, one potential methodology which could be suitable to evaluate courtroom accommodations is a matched control group design (Komro et al., 2016). By implementing different sets of accommodations at different locality courts with similar populations, this would enable the determination of the most effective set of accommodations. There are many other ways to frame such an evaluation, and useful explorations of these have been found in other community settings (Komro et al., 2016; Mikkelsen et al., 2016). The foundation of any evaluation would be selecting appropriate measures, such as of procedural fairness, which could be operationalised in an interview format or through a survey design. There are multiple different options available regarding the measurement of procedural fairness in courtrooms (e.g., LaGratta & Jensen, 2015), however it must be noted that these themselves would need to be altered significantly to be accessible to young adults with neurodivergence. When looking to evaluate the effectiveness of training, measurement of both young adult and criminal justice workers' perception of staff knowledge, confidence, and application of knowledge would be helpful. Future research and implementation of courtroom accommodations for neurodivergent young adults should look to embed an ongoing developmental evaluation – which involves an evolving set of accommodations – into practice to ensure true access to justice.

## 5 CONCLUSION

During the environmental assessment, focus group assessors recognised significant signs of potential neurodivergence amongst the group of young adults, indicating a clear need for systems such as the YAL initiative. They also recommended neurodiversity training, trauma‐informed education, and screening for neurodivergence in order to maximise the potential of the YAL and similar initiatives internationally. Accommodations were identified which could be applied in the context of limited resources, as well as more aspirational strategies requiring greater resource commitment. Importantly, different courts may require different framing of accommodations depending on the population and community they serve and accommodations must be responsive to such diversity. A tiered accommodation model would greatly improve inclusion whilst maintaining feasibility for criminal justice systems, and ensuring that those who need more individualised support receive it. The range of opportunities where improvements have been recommended, will be aided most significantly by neurodiversity training of the judiciary and courtroom officials that frames neurodiversity from a contemporary perspective. The strategies identified in this article aim to increase engagement, understanding, and empower young adults with neurodiversity to feel heard and understood following their time in court. Ultimately, the legal system has a clear opportunity to reduce societal barriers for young adults with neurodivergence, and be a source of social justice reform which, in turn, could greatly improve outcomes both for young adults, their families/whānau, and society as a whole.

## Supporting information

Supplementary Material

## Data Availability

Data sharing is not applicable to this article as no new data were created or analysed in this study.
