# Supplementary material for: Responding to neurodiversity in the courtroom: A brief evaluation of environmental accommodations to increase procedural fairness
Source: Crim Behav Ment Health. 2022 Aug 5;32(3):197–211. doi: 10.1002/cbm.2239 (PMC9540328; doi:10.1002/cbm.2239)
Supplement: Supplementary file 1 — Supplementary Material [file CBM-32-197-s001.pdf]

APPENDIX 1 Key criteria for observational assessment of YALC: panel guidance sheet

Questions to answer from a neurodiversity accessibility perspective relating to each of the aspects below include:

- What works well?
- Any additional accommodations or recommendations for court accessibility?
- Any accessibility barriers to participation?

Current court elements	Things to consider during observation:	
**Courtroom layout**	Size of space	
	Location and physical attributes of dock	
	Any other key physical attributes of courtroom	
	Number of other people present	
	Location and accessibility of judge	
	Location and accessibility of defence lawyers	
	Location and accessibility of support agencies	
	Location and accessibility of family/whānau	
**Active engagement**	Introduction to courtroom	

APPENDIX 1 Key criteria for observational assessment of YALC: panel guidance sheet

Questions to answer from a neurodiversity accessibility perspective relating to each of the aspects below include:

- What works well?
- Any additional accommodations or recommendations for court accessibility?
- Any accessibility barriers to participation?

**from young person**		
	Use of own voice	
	Level of young person's understanding	
	With the judge	
	With whānau/support person	
	With defence lawyer	
	With multi-disciplinary team e.g. Police Prosecutor	
**Multi-disciplinary team**	Location of multi-disciplinary team	
	Accessibility of multi-disciplinary team	

APPENDIX 1 Key criteria for observational assessment of YALC: panel guidance sheet

Questions to answer from a neurodiversity accessibility perspective relating to each of the aspects below include:

- What works well?
- Any additional accommodations or recommendations for court accessibility?
- Any accessibility barriers to participation?

	Engagement of multi-disciplinary team	
	Comprehensiveness of multi-disciplinary team	
**Language accessibility**	Judge	
	Defence lawyer	
	Support staff	
**Information booklet**	Language accessibility	
	Time to read	
	Any support given	
**Other criteria related to neurodiversity to consider during observation:**		

APPENDIX 1 Key criteria for observational assessment of YALC: panel guidance sheet

Questions to answer from a neurodiversity accessibility perspective relating to each of the aspects below include:

- What works well?
- Any additional accommodations or recommendations for court accessibility?
- Any accessibility barriers to participation?

**Other factors related to engagement**	Introduction to courtroom	
	Use of own voice	
	Level of understanding	
	Judge	
	Defence lawyer	
	Support staff	
**Presentation**	Young person's presentation during session<br>(e.g. engagement, dress, mental state, acknowledgement of others)	
	Presentation of other court attendees during session	

APPENDIX 1 Key criteria for observational assessment of YALC: panel guidance sheet

Questions to answer from a neurodiversity accessibility perspective relating to each of the aspects below include:

- What works well?
- Any additional accommodations or recommendations for court accessibility?
- Any accessibility barriers to participation?

	(e.g. engagement, dress, acknowledgement of others)	
	Responsiveness of court to young person's presentation during session	
**Signage**	Accessibility	
	Placement	
	Any support given	
**Distraction factors**	Background noise	
	Other potentially disruptive stimuli	
